# Parotid Ewing's sarcoma: Extra-skeletal uncommon condition

**DOI:** 10.1016/j.amsu.2021.102304

**Published:** 2021-04-13

**Authors:** Ayoub Sabr, Rachid Aloua, Ouassime Kerdoud, Faiçal Slimani

**Affiliations:** a Faculty of Medicine and Pharmacy, Hassan II University of Casablanca, B.P 5696, Casablanca, Morocco; b Oral and Maxillofacial Surgery Department, CHU Ibn Rochd, B.P 2698, Casablanca, Morocco

**Keywords:** Ewing sarcoma, Extra-skeletal ewing sarcoma, Parotid, Prognosis

## Abstract

**Introduction:**

The parotid gland is a very rare location of Ewing's sarcoma. Its clinical and radiological manifestations are atypical. Its management is based on a multidisciplinary approach to improve prognosis and survival.

**Discussion:**

The diagnosis of parotid Ewing's sarcoma is determined by a combination of genetic and histological criteria. Its local aggressiveness and high metastatic potential require prompt and appropriate management. Many prognostic features affect its management, the most important is the TNM staging.

**Conclusion:**

Limited cases of parotid Ewing's sarcoma have been published in the literature. This small number of reported cases is the only way to study its characteristics.

## Highlights

• The parotid gland is a very rare location of Ewing's sarcoma.
• The diagnosis of parotid Ewing's sarcoma is determined by a combination of genetic and histological criteria.
• Many prognosis features affect its management, the most important is the TNM staging.

## 1 Introduction

Extra-skeletal Ewing's sarcoma is a rare soft tissue cancer that can develop in any part of the body [2]. Parotid location is very rare and of uncommon clinical and radiological manifestation [2,5]. A genetic predisposition is linked to the appearance of this type of sarcoma [1,4]. Management is multidisciplinary, based on surgery combined with adjuvant radiochemotherapy for the prevention of local recurrence and to improve the long-term prognosis [1,2,5].

This case has been reported in line with the SCARE criteria [9].

## 2 Case report

43-year-old female patient who had surgery for right breast cancer in 2015 and underwent a right mastectomy followed by 5 sessions of radiotherapy and 6 sessions of chemotherapy stopped in August 2016, and on hormonal therapy (Tamoxifen 1cp/d), presented to our department of maxillofacial surgery in November 2020 for left parotid swelling that had been progressing for 3 months. The patient was reported to our department by a regional referral hospital.

The clinical examination revealed a parotid mass of 4 cm long axis with skin that appeared to be normal in appearance, firm on palpation, painless, mobile to the superficial and deep plane, without facial palsy, with moderate trismus and mobile high homolateral jugulo-carotid cervical adenopathy with no visible inflammatory signs, an examination of the orifice of the Stenon's duct shows no inflammation or bloody outflow, and otoscopic examination finds no signs of invasion of the external ear duct.

**Fig. 1:**
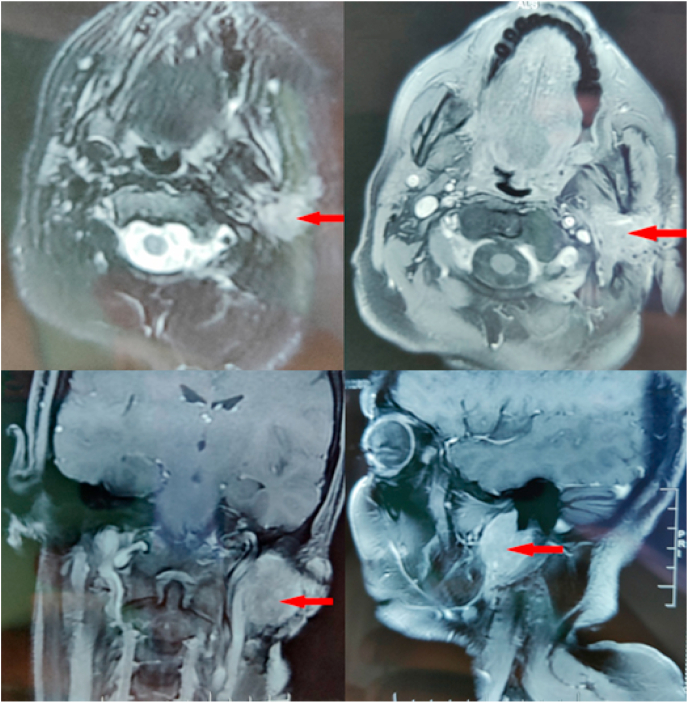
Facial MRI showing a left parotid tumor in isosignal T1 and T2.

Facial MRI revealed a slightly limited tissue process in the left parotid gland with polylobed contours in the T1 isosignal and T2 isosignal, which was heterogeneously enhanced after injection of the contrast agent [Fig. 1], with thickening and infiltration of the opposite soft tissues in contact with the medial pterygoid muscle and the external jugular vein, without any damage to the mandibular bone at the complimentary scan.

**Fig. 2:**
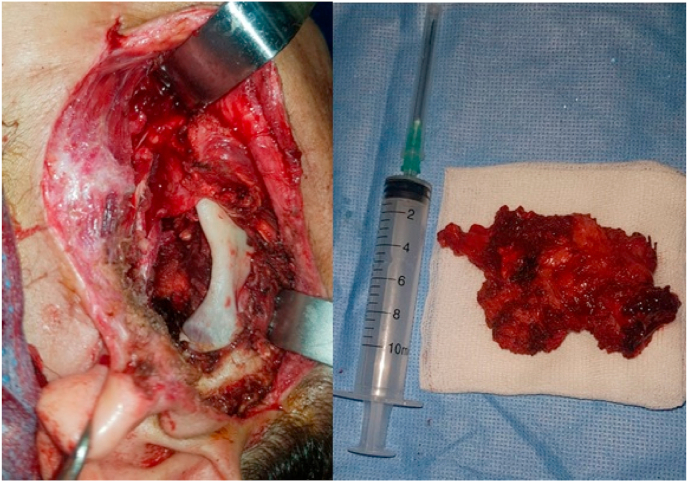
Surgical field after total left parotidectomy with preservation of the facial nerve + Surgical specimen.

The extension survey did not reveal any distant metastasis. The patient underwent exotic parotidectomy with extemporaneous examination revealing carcinoma, followed by full parotidectomy with facial nerve preservation and homolateral supra-digastric adenectomy [Fig. 2]. Surgical intervention was performed by our chief of maxillofacial department who has 15 years of operative experience.

The anatomopathological outcome of the specimen concluded at parotid Ewing's sarcoma with the positivity of CD99, cytokeratin AE1AE3, and synaptophysin. The patient was sent to oncology for adjuvant radiotherapy then was followed after her discharge in the outpatient department by a rhythm of twice a month for two first months and then once per month for a year.

## 3 Discussion

Extra-skeletal Ewing's sarcoma is a rare malignant tumor of mesenchymal cellular origin with histological characteristics similar to those of bony Ewing's sarcoma [2,6]. It can affect the soft tissues of all parts of the body with predominance in the chest wall, pelvis, pleural cavities, and paravertebral region [1,2,5]. It is characterized by its locoregional aggressiveness and high metastatic potential. The cervicofacial location is very rare and few cases have been published in the literature [5,8].

Ewing's sarcoma occurs mainly among adolescents and young adults aged 10–30 years without gender predominance [8]. Etiopathogenically, it is a mesenchymal tumor related to cerebral neuroectodermal tumors [1,2,4,5] with genetic abnormalities such as t(11;22)(q24;q12) reciprocal translocation in 80% of cases and other rare types of translocations t(21;22)(q22;q12), t(7;22)(p22;q12), t(17;22)(q12;q12) and t(2;22)(q33;q12) in the remaining cases [2,3,5,6]. In the different forms of translocations found in Ewing's sarcoma of soft tissue, the abnormal protein resulting from the expression of the fusion genes causes continuous activation of the membrane IGF-1 receptor responsible for tumor proliferation [2,5].

The clinical manifestations of Ewing's sarcoma of the parotid gland are non-specific and include swelling in the rapidly developing parotid region, trismus, locoregional pain, peripheral facial paralysis, and homolateral cervical lymphadenopathy [2,7]. Imaging, especially high-resolution CT and MRI scans, is useful in the diagnosis and assessment of extension, although the characteristics are not specific. In our case, the patient did not present with facial palsy, which means that there was no invasion of the facial nerve by the tumor. On MRI, these tumors are hyposignal T1 and hypersignal T2, rising heterogeneously after contrast [2]. In our case, the tumor was isosignal T1 and isosignal T2 on MRI, which proves that this type of tumor has an inconsistent radiological presentation. Remote metastases are dominated by pulmonary, bone, and cerebral localization [2].

The definitive diagnosis of parotid Ewing's sarcoma is histological due to its specific clinical and radiological characteristics. Histologically it is a small round cell tumor with a positive marker CD99/MIC2 in immunohistochemistry [1,2,[5], [6], [7]], in our case, the immunohistochemical study was positive for CD99, cytokeratin AE1AE3, and synaptophysin.

The management of parotid Ewing's sarcoma is multidisciplinary, including surgeons and oncologists [1]. Surgical excision with adjuvant radiotherapy is indicated for localized and readily operable forms of the tumor [1,2,7]. Neoadjuvant chemotherapy is indicated for the reduction of tumor size for large localized tumors [1]. In our case, there was no invasion of noble anatomical structures contraindicating surgery, the management was based on surgery by performing a total conservative parotidectomy of the facial nerve with complementary adjuvant radiotherapy.

The most important prognostic factors are represented by tumor size and the presence of distant metastases at the time of diagnosis, which influences the modalities and results of treatment [5].

## 4 Conclusion

Parotid Ewing's sarcoma is a rare entity of this kind of tumor which is characterized by its locoregional aggressiveness and high metastatic potential. Its therapeutic management can present a challenge to the treating physician, but a multidisciplinary approach including surgery, radiotherapy, and chemotherapy when indicated can lead to better long-term results.

## Ethical approval

Written informed consent was obtained from the patient for publication of this case report and accompanying images. A copy of the written consent is available for review by the Editor-in-Chief of this journal on request.

## Sources of funding

The authors declared that this study has received no financial support.

## Author contribution

Ayoub Sabr: Corresponding author writing the paper.

Rachid Aloua: writing the paper.

Ouassime kerdoud: writing the paper.

Faiçal Slimani: Correction of the paper.

## Registration of research studies

1. Name of the registry:

2. Unique Identifying number or registration ID:

3. Hyperlink to your specific registration (must be publicly accessible and will be checked):

## Guarantor

Ayoub Sabr.

## Consent

Written informed consent was obtained from the patient for publication of this case report and accompanying images. A copy of the written consent is available for review by the Editor-in-Chief of this journal on request.

## Provenance and peer review

Not commissioned, externally peer reviewed.

## Declaration of competing interest

Authors of this article have no conflict or competing interests. All of the authors approved the final version of the manuscript.
